# Enzymatic properties and biological activity of resuscitation-promoting factor B of *Rhodococcus* sp. (GX12401)

**DOI:** 10.3389/fmicb.2022.965843

**Published:** 2022-10-05

**Authors:** Xu Gong, Huijiao Lu, Jiafa Wu, Yan Zhou, Lifang Yang, Yibing Wang, Naikun Shen, Mingguo Jiang

**Affiliations:** ^1^Guangxi Key Laboratory of Polysaccharide Materials and Modification, School of Marine Sciences and Biotechnology, Guangxi Minzu University, Nanning, China; ^2^School of Chemistry and Chemical Engineering, Guangxi Minzu University, Nanning, China

**Keywords:** resuscitation-promoting factor, *Rhodococcus* sp., muralytic activity, VBNC, resuscitation

## Abstract

Resuscitation-promoting factor B (RpfB) is one of the five members of Rpf-like family in *Mycobacteriales*, which have the resuscitation-promoting activity. Most strains of *Rhodococcus* also have RpfB gene, but the study of *rpfB* gene in *Rhodococcus* is not thorough. Here, we amplified the *rpfB* gene of intact *Rhodococcus* sp. (GX12401) and cloned it into pET30a (+) expression vector. Then a recombinant form of soluble RpfB was expressed in *Escherichia coli* BL21. The soluble recombinant RpfB was purified by Ni–Sepharose affinity chromatography and molecular weight of the protein was 55 kDa, determined by 12% SDS–PAGE stained with Coomassie brilliant blue R-250. When 4-methylumbelliferyl-β-D-N,N′,N″-triacetylchitoside was used as enzyme substrate to test lysozyme activity, the recombinant protein RpfB had good stability and enzyme activity, and the lysozyme activity was low (4.74 U), among which Mg^2+^, Na^+^, Al^3+^ and DMSO could significantly increase the activity of RpfB. The purified recombinant protein was added to Rhodococcus VBNC cells, and the VBNC cells were resuscitated at the concentration of 1 picomolar concentrations, which increased by 18% compared with the control, while the cell resuscitation was inhibited at the concentration of 1,000 picomolar concentrations. Therefore, RpfB can improve the survival ability of *Rhodococcus* in extreme or harsh environment and enhance the corresponding biological activity.

## Introduction

Resuscitation-promoting factor (Rpfs) was discovered in the supernatant of *Micrococcus luteus* in 1998 (Mukamolova et al., 1998). At present, rpfs is the most studied resuscitation stimulator because of its mild conditions for resuscitating viable but non-culturable (VBNC) cells. It is a highly conserved protein and promotes the growth rate of culturable cells more than 100 times, and has a growth-promoting effect on a variety of Gram-positive bacteria (Su et al., 2013). In the past decades, more than 30 Rpf genes have been found in different microorganisms, while they belong to the same protein family, but there are some differences. *Mycobacterium bovis* and *Mycobacterium tuberculosis* produce five kinds of Rpf proteins, namely Rpf A ~ E (Mukamolova et al., 2002). Protein RpfB is essential for VBNC cells to resuscitate *M. tuberculosis* because lacking of RpfB protein will lead to the inability of *M. tuberculosis* VBNC cells recovery (Tufariello et al., 2006). *Streptomyces coelicolor* can produce five kinds of Rpf proteins, all of which are secretory proteins, which are different from Rpf proteins of *M. tuberculosis* (Sexton et al., 2015). There are two genes *rpf* 1 and *rpf* 2 in *Corynebacterium glutmicum*. When the single gene was mutated, the cell growth rate did not change significantly. The double gene mutation caused the growth retardation of the strain and made *C. glutmicum* VBNC cells unable to recover (Hartmann et al., 2004). Studies have shown that the expression of the *rpf* 2 gene in *C*. *glutmicum* belongs to triple transcriptional regulation and is controlled by the regulators GlxR, RamA and RamB (Jungwirth et al., 2008). The Rpf protein isolated and purified from *R. erythropolis* can promote the recovery of VBNC cells of *R. erythropolis*. In addition, it was determined that the amino acid site Gln69 of Rpf protein is related to the wall-lytic activity (Luo et al., 2019). According to amino acid sequence analysis of Rpf proteins, they belong to the family of hydrolytic transglycosylases with a lysozyme-like fold (Mukamolova et al., 2006), and the purified recombinant His-tagged *M. luteus* Rpf was demonstrated to possess muralytic activity, capable to hydrolyze both hydrolyzing fluorescamine-tagged *M. luteus* cell walls and synthesized Lysozyme substrate 4-methylumbelliferyl-β-D-N,N′,N″-triacetylchitoside (Tufariello et al., 2006). The Rpf protein also has weak proteolytic activity against N-CBZ-Gly-Gly-Arg-β-naphthylamide, a substrate for trypsin-like enzymes (Telkov et al., 2006). The Rpf proteins are widely distributed in Gram-positive bacteria of the *Actinobacteria phylum* and biological activity (resuscitation or growth promotion) has been demonstrated for several representatives (Punta et al., 2012), including all five proteins found in *M. tuberculosis* and its close relatives (Zhu et al., 2003; Mukamolova et al., 2006). At present, there are many research on Rpf protein of *M. tuberculosis*, but few on Rpf protein of *Rhodococcus*. It has been reported that *Rhodococcus* secreting Rpfs can promote the resuscitation of many strains, and we found that number of *Rhodococcus* contain Rpfs through bioinformatics analysis, which means that *Rhodococcus* is one of the important sources of Rpfs. Through heterologous expression and fermentation, more culturable microorganisms were isolated and purified, and the utilization rate of microbial resources was improved (Su et al., 2015a,b, 2018, 2019).

The research on the formation and recovery mechanisms of viable but non-culturable (VBNC) microorganisms has always been a hot and difficult point in the field of microorganisms. Viable but non-culturable status was discovered in *Escherichia coli* and *Vibrio cholerae* in 1982 and was later considered to be the result of gene regulation (Xu et al., 1982; Ravel et al., 1994). Currently, the VBNC state has been regarded as a survival strategy for microorganisms to defend against adverse living environments; in this state, bacteria cannot grow on routine culture media but their metabolic activity can still be detected (Dong et al., 2020). Under good living conditions, some bacteria in the VBNC state can be restored to the culturable state, and some bacteria gradually move towards apoptosis, seriously affecting the application of functional bacteria in a specific environment. Therefore, it is of great significance to study the mechanism of VBNC state formation and its recovery to reveal the existing mechanism of microbial VBNC.

In this study, a *Rhodococcus* sp. (GX12401) producing RpfB was obtained from mangrove sediment after enrichment culture. The supernatant is separated from the strain fermentation, and the crude enzyme properties are analyzed. The *rpfB* gene was adjusted in accordance with the *Rhodococcus* sp. (GX12401) of the genomic database, and the total length of the *rpfb* gene fragment was constructed for the establishment of the engineered strain. The gene is sequence alignment compared to other *rpfB* genes, understanding the evolutionary relationship, and performs a biological analysis of the RpfB sequence, preliminary understanding of the properties of the protein. The recombinant protein RpfB was purified by nickel column and its enzymatic properties were studied, which provided a basis for screening new functional strains and studying the mechanism of reviving VBNC. In addition, we used the antibiotic ciprofloxacin to induce *Rhodococcus* sp. (GX12401) to enter VBNC state. When the recombinant protein RpfB is 1 picomolar concentrations, it can recover its own VBNC state, and when it is 1,000 picomolar concentrations, it will inhibit its own recovery.

## Materials and methods

### Materials

We obtained DNA polymerase, *Bam*HI and *Hind*III restriction endonuclease, T4 ligase, TIAN amp Bacteria DNA Kit, TIAN prep Mini Plasmid Kit, Universal DNA Purification Kit, and EasyPure Quick Gel Extraction Kit., Ltd. from TRAN (Beijing, China). Ni-NTA agarose was obtained from Qiagen Gmbh (Hilden, Germany). Fluorogenic glycanase substrate of 4-methylumbelliferyl-β-D-N,N′,N″-triacetylchitotrioside (4-MUF-3-NAG) was obtained from Sigma-Aldrich, Co., (St. Louis, MO, United States). CFDA SE Cell Proliferation Fluorescent Probe was purchased from Sangon Biotech Co., Ltd. (Shanghai, China). Host cell strains, *E. coli* TOP10 and *E. coli* BL21, were obtained from TransGen Biotech CO., Ltd. (Shanghai, China) and the pET-30a (+) was purchased from Sangon Biotech Co., Ltd. (Shanghai, China). All other chemicals used in this study were of analytical grade. The *Rhodococcus* sp. (GX12401) was reserved in the China General Microbiological Culture Collection Center (CGMCC) under the number CGMCC NO.23759.

### Bacterial strains and culture conditions

The mangrove sediment samples were stored at 28°C for 1 week and ground into powder. 2.0 g of each sample was dried in the oven for 2 h (80°C), and added to 5 ml aseptic 0.1%Na_4_P_2_O_7_ solution. Then, a few glass beads were added to the solution. The sediment sample suspension was obtained by shaking in the shaker at the rate of 180 rpm for 2 h. 1 ml of sediment suspension was diluted for 4 times, and the sediment sample dilution with a concentration of 10^−4^ was obtained. 50 μl of diluted solution was diluted and coated on the separation plate, and the experiments were repeated 3 times in each group. After the procedures of isolation, purification, and 16S rRNA identification, a strain of *Rhodococcus* sp. (GX12401) was obtained and stored in a 30% glycerol cryopreservation tube (−80°C; Shi et al., 2018). The *Rhodococcus* sp. (GX12401), *E. coli* TOP10 and *E. coli* BL21 strains were cultivated in sterile Luria-Bertani (LB) medium at pH 7.4 ± 0.2, containing (g/L): tryptone 10.0, yeast extract 5.0, NaCl 10.0, and agar powder 15.0 (for solid culture medium).

### Plasmids and cloning of the *rpfB* gene

Oligonucleotide primers used for the cloning of *rpfB* gene from *Rhodococcus* sp. (GX12401) were designed from the genomic database of *Rhodococcus* sp. (GX12401) PRJNA785756. The nucleotide sequence of *rpfB* gene was deposited in GenBank with accession number ON357679. DNA was obtained from Chelex-100 and used as the template for polymerase chain reaction (PCR) amplification. The gene encoding *rpfB* was amplified using *Pfu* DNA polymerase with primer pairs: 5′-CGCGGATCCATGTCACCTTTCACCAAGATCAA-3′ and 5′-CCCAAGCTTGCGCAGACCGAGCTTGCTGG-3′ (underlining indicates the added *Bam*HI and *Hind* III sites). The PCR reaction condition was as follows: 95°C for 10 min, one cycle; 95°C for 1 min, 59.6°C for 1 min, 72°C for 1 min 30 s, 30 cycles; with a final extension at 72°C for 10 min. The PCR product was purified and ligated into the pUCm-T vector to obtain the pUCm-*rpfB* plasmid. The pUCm-*rpfB* plasmid was purified and digested with *Bam*HI and *Hind*III, it was then inserted into the pET-30a (+) vector digested with the same enzymes. The constructed ligation product pET-30a (+)-*rpfB* was transformed into *E. coli* TOP10 and verified using automated DNA sequencing.

### Bioinformatic analyses

The open reading frame (ORF) and encoded amino acid sequence of RpfB were analyzed using ORF Finder^1^ followed by translation of the nucleotide sequence using DNAMAN software. Domain structure was analyzed at the National Center for Biotechnology Information (NCBI)^2^ and the three-dimensional structure of RpfB was predicted using SWISS-MODEL (Ruggiero et al., 2016).^3^ A phylogenetic neighbor-joining tree was constructed using MEGA-X software, and ESPript 3.0 was used for sequence alignment.^4^

### Expression and purification of *rpfB*

For the expression of recombinant RpfB, pET-30a (+)-*rpfB* was transformed into *E. coli* BL21 cells. Cells were cultured at 37°C in a liquid LB medium containing 50 mg/L Kanamycin Sulfate. The cells were kept at 4°C for 0.5 h when OD600 reached 0.4 ~ 0.5, and then 0.2 mM isopropyl β-D-galactosamine (IPTG) was added to induce the expression, after which cells were incubated at 20°C for a further 16 h. Protein expression was checked by sodium dodecyl sulfate-polyacrylamide gel electrophoresis (SDS-PAGE; 12% separation gel, 4% concentrated gel). The 50 ml of bacterial broth was placed in centrifuge tubes and centrifuged at 8,000 rpm for 5 min at 4°C, and the bacterial cells were collected and re-suspended in 10 ml of lysis buffer (pH 7.4, 50 mM sodium phosphate, 10 mM imidazole, 300 mM NaCl). The mixture was then sonicated with ultrasonic cell disrupter on ice bath (power of 250 ~ 300 W, work 3 s, interval 7 s, 150 cycles). The broken cell solution was then centrifuged at 8,000 rpm for 10 min at 4°C. The supernatant was collected and filtered through a 0.22 μm sterile filter. The supernatant, comprising crude protein extracts containing RpfB, was immediately applied to a 5 ml Ni-NTA affinity chromatography column pre-equilibrated with binding buffer (pH 7.4, 50 mM sodium phosphate, 20 mM imidazole, 300 mM NaCl) at 4°C. During the protein binding period, they mixed at intervals of 15 min and the binding time was 1 h. The column was washed with 50 ml of washing buffers (pH 7.4, 50 mM sodium phosphate, 50 mM imidazole, 300 mM NaCl, then pH 7.4, 50 mM sodium phosphate, 80 mM imidazole, 300 mM NaCl). RpfB was eluted with 50 ml elution buffer containing (pH 7.4, 50 mM sodium phosphate, 200 mM imidazole, and 300 mM NaCl, respectively). Purity of the recombinant protein was analyzed using 12% SDS-PAGE with Coomassie Brilliant Blue R-250 staining. The molecular mass of the purified enzyme was determined by comparison with protein molecular weight markers. The eluted fractions which contained target protein were concentrated and exchanged into a 50 mM Acetate-acetate buffer solution (50 mM CH₃COONa, pH 7.0), pH 7.0, using 30 kDa centrifugal filter units, and enzyme preparations were stored at 4°C until use.

### Muralytic activity analysis of the purified recombinant proteins

Similar to previous reports, we characterized the RpfB protein as having muralytic activity by decomposing an artificial fluorescent substrate 4-methylumbelliferyl-β-D-N,N′,N″-triacetylchitotrioside (4-MUF-3-NAG; Mukamolova et al., 2006). Five mg/ml of 4-MUF-3-NAG solution was prepared with pyridine: water (1:1) and diluted into 100 mM working solution with 50 mM sodium acetate buffer. Briefly, 50 μl of 50 mM Acetate-acetate buffer solution (50 mM NaAc, pH 7.0, containing 100 mM 4-MUF-3-NAG) was added to 150 μl of the recombinant protein (0.20 mg/ml), the mixture was then incubated at 40°C for 7 h. The fluorescence intensity was detected with Varioskan LUX Multimode Microplate Reader using excitation wavelength of 360 nm and a read-out of 455 nm. Under the conditions of this laboratory, the standard curve of 4-MUF is *y* = 18,094*x* + 723.43, and the production of 1.0 nM of product enzyme per milliliter of incubated sample (protein) per hour is regarded as an enzyme activity unit (Luo et al., 2019). Therefore, the conversion formula of enzyme activity unit (U) is: *U* = [((*A*2–*A*1)/*T*)/*y*]*1000*5, where A1 is the fluorescence value at the beginning of the reaction, *A*2 is the fluorescence value at the end of the reaction, and *T* is the reaction time (*h*), and *y* is the fluorescence value of 4-MUF.

The optimum temperature for RpfB muralytic activity was determined by incubating enzyme preparations with 4-MUF-3-NAG at various temperatures between 10°C and 50°C in 50 mM Acetate-acetate buffer at pH 7.0, and the experiment was incubated for 7 h, and each group was repeated three times. The effects of metal ions and chemical reagents (Ni^2+^, Cu^2+^, Co^2+^, Mn^2+^, Mg^2+^, Na^+^, Zn^2+^, Li^+^, K^+^, Al^3+^, NH^+^, Tris, and SDS) on RpfB muralytic activities at final concentrations of 100 mM were also investigated, as were the effects of organic solvents and reagents [methanol, ethanol, isopropanol, glycerin, isoamyl alcohol, Tween 80, acetic acid, chloroform, dimethylsulfoxide (DMSO), and β-mercaptoethanol] at final concentrations of 1% (*v*/*v*). Residual RpfB muralytic activities were measured in standard assay conditions and each group was repeated three times.

### Induction of VBNC cells

The strain *Rhodococcus* sp. (GX12401) was used in this study and preserved in 30% glycerol (*v*/*v*) at −80°C. Prior to use, bacterial cells were grown in a sterile LB medium on a shaker incubator (200 rpm) at 30°C for 24 h. Then the cells were inoculated in (1%, *v*/*v*) LB medium under the same conditions until grown to the exponential phase. Ciprofloxacin was added to the exponential phase cells at a final of 64 mg/L. The cells were cultured in the dark on a shaker incubator (200 rpm) at 30°C for days to induce the VBNC state. All experiments were performed in triplicates. LB agar plates were used to analyze each sample obtained at different times by flow cytometer.

### Culture and viability study

The culturable cells were counted using the standard plate count method. Each sample was serially diluted 10-fold with 0.9% (*w*/*v*) physiological saline (sodium chloride solution) and incubated for 48 h on LB agar medium at 30°C. The culturable cell counts of *Rhodococcus* sp. (GX12401) were calculated as counts per milliliter (CFU/ml). In order to detect the changes of enzymatic activities of VBNC cells and logarithmic cells, we used API ZYM kit to determine the enzymatic activities in 19 according to the manufacturer’s instructions. The viable cell number was measured using a flow cytometer (Su et al., 2015a). By using HHBS buffer, 0.5 μM CFDA SE fluorescent dye was prepared, *Rhodococcus* sp. (GX12401) in VBNC state was added to CFDA SE stain, adjusted 1 ~ 5 × 10^5^ cells/ml, and cells were cultured under 37°C for 15 min. The labeled suspension tubes were centrifuged at 1,000 rpm for 5 min and the supernatant was removed. Cells were rinsed two times with Hepes buffer (HHBS, 1.26 mM CaCl_2_, 0.49 mM MgCl_2_·6H_2_O, 0.41 mM MgSO_4_.7H_2_O, 5.33 mM KC1, 0.44 mM KH_2_PO_4_, 4.17 mM NaHCO_3_, 137.93 mM NaCl, 0.34 mM Na_2_HPO_4_, 5.56 mM D-Glucose, 20 mM Hepes) and cells were re-suspended in 500 μl pre-warmed HHBS buffer. Fluorescence change was monitored with a flow cytometry or fluorescence microscope at Ex/Em = 490/520 nm.

## Results

### Expression and purification of RpfB

**Figure 1:**
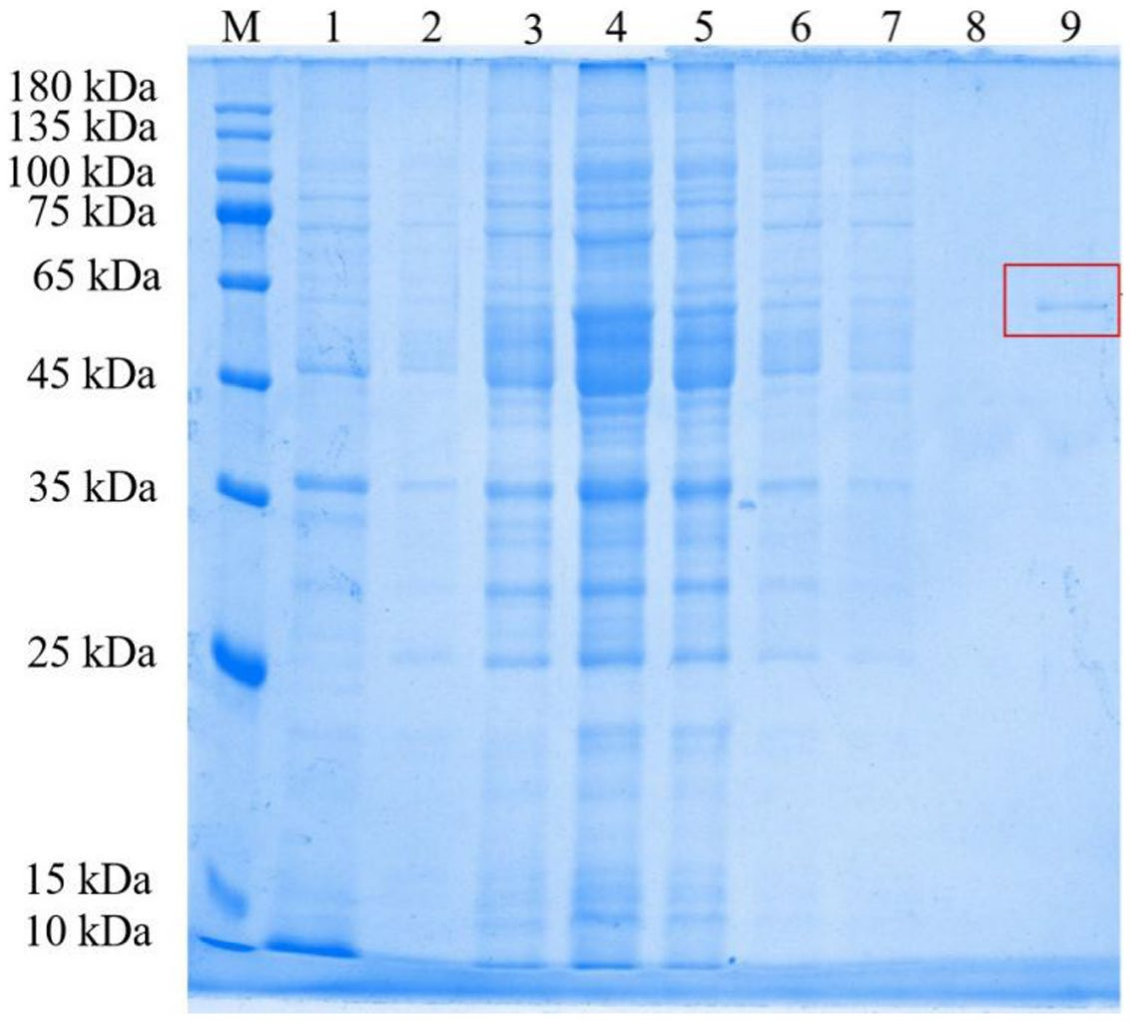
Representative gel of purification steps of RpfB protein from *Rhodococcus* sp. (GX12401). M, Protein molecular weight markers; 1, *Escherichia coli* BL21 cleavage protein containing pET30a (+); 2, Bacterial proteins before induction; 3, Insoluble body protein; 4, Soluble protein; 5, Unbound protein; 6, Protein eluted with buffer containing 20 mM imidazole; 7, Protein eluted with buffer containing 50 mM imidazole; 8, Protein eluted with buffer containing 80 mM imidazole; 9, Proteins were eluted with buffer containing 200 mM imidazole, and protein bands were marked with red boxes. The gel was stained with Coomassie Brilliant Blue R-250.

The gene encoding *rpfB* was successfully cloned from *Rhodococcus* sp. (GX12401) and expressed in *E. coli* BL21 and purified. The molecular weight of the protein measured by SDS-PAGE is 55 kDa (Figure 1), which is not consistent with the expected 46.6 kDa of the translated nucleotide sequence. The relative molecular weight of the actual protein is larger than the theoretical value 8.4 kDa, which is speculated to be due to the upward shift caused by the His-tag on the carrier (Tang et al., 2000). Therefore, we identified the purified band by in-flight mass spectrometry and finally determined it to be the target protein.

### Bioinformatic analysis

**Figure 2:**
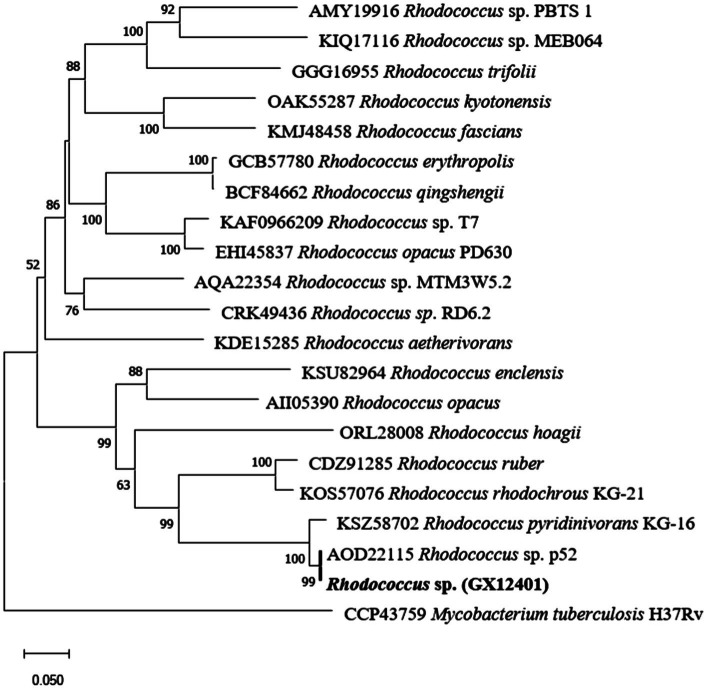
The Neighbor-joining (NJ) tree based on the *Rhodococcus rpfB* gene sequences showing the phylogenetic relationship of strain *Rhodococcus* sp. (GX12401) with related taxa. The tree was rooted by the mid-point method. The sequence of *M. tuberculosis* H37Rv (CCP43759.1) was used as an outgroup. Bootstrap support values were calculated from 1,000 replicates and only values above 50% are shown. Bar, 0.05 substitutions per nucleotide position.

The gene encoding *rpfB* included a complete ORF, encoding a protein of 378 amino acids. Domain structure analysis revealed *rpfB* to contain a transmembrane domain, three DUF 348, a G5, and a transglycosylase. The enzyme was identified and belongs to *rpfs* family. Sequence alignment using BLASTP software (NCBI) showed that *Rhodococcus* sp. (GX12401) had 99.20% sequence identity and 100% sequence coverage with *Rhodococcus* (WP_006553818.1), followed by *Rhodococcus* sp. HS-D2 (WP_064255739.1) with 98.93% identity and 100% sequence coverage. In order to understand the evolutionary relationship of *rpfB* gene in *Rhodococcus*, we collected all *Rhodococcus* strains with *rpfB* gene through NCBI and excluded the repetitive strains. We constructed phylogenetic tree of RpfB protein of genus *Rhodococcus*, with *M. tuberculosis* H37Rv (CCP43759.1) as out group Figure 2. RpfB protein is most similar to *Rhodococcus* sp. P52 (AOD22115), followed by *Rhodococcus* sp. KG-16 (KSZ58702).

**Figure 3:**
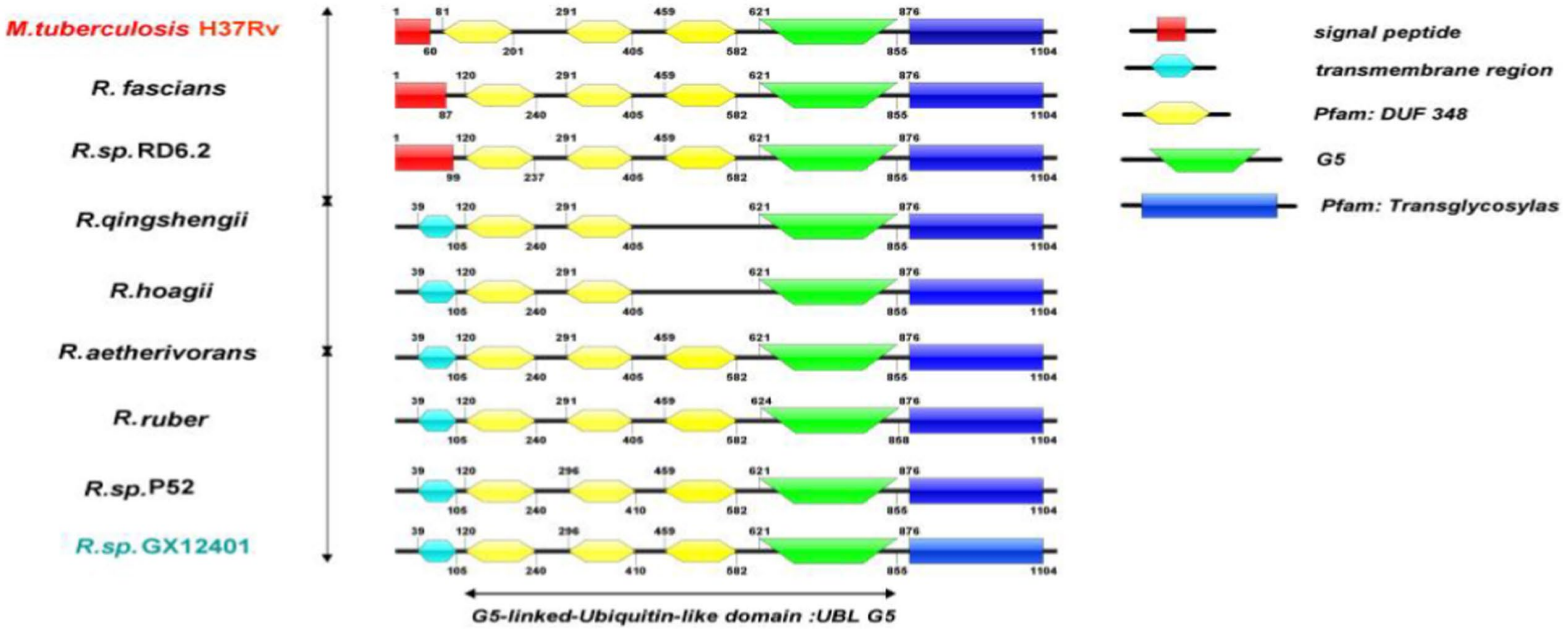
Domain structure of RpfB protein of genus *Rhodococcus.*

**Figure 4:**
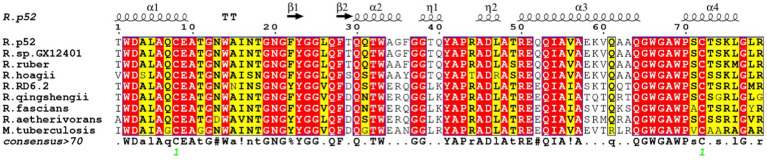
Alignment of amino acids between *Rhodococcus* and *M. tuberculosis* H37Rv; the same amino acids are expressed in capital letters corresponding to amino acids; highly conserved amino acids are expressed in red text; low conserved amino acids are expressed in yellow text; amino acid sequence number: *Rhodococcus* sp. P52 (AOD22115.1); *R. ruber* (CDZ91285.1); *R. hoagii* (ORL28008.1); *R. RD 6.2* (CRK49436.1); *R. qingshengii* (BCF84662.1); *R. fascians* (KMJ48458.1); *R. aetherivorans* (KDE15285.1); *M. tuberculosis* H37Rv (CCP43759.1).

In this study, the representative strains of *Rhodococcus* were compared with *M. tuberculosis* H37Rv (CCP43759.1). Through the Espript 3.0 sequence display tool, it was found that the two domains of G5 and transglycosylase were highly conserved, and highly conserved regions were shown in Figure 3. In addition, according to the different domains, the RpfB proteins of different strains of the same genus are different. RpfB contains a complete domain, *R. qingshengii* and *R. hoagii* lack a DUF348 domain, but the remaining domains are arranged at positions unchanged, as shown in Figure 4. In addition, *R. fascians*, *Rhodococcus* sp. RD6.2 and reference strain *M. tuberculosis* H37Rv all have signal peptides but not transmembrane domains, while most RpfB proteins also have transmembrane domains, which is helpful for us to further investigate physical and chemical properties and resuscitation mechanism of RpfB proteins.

### Muralytic activity

Through the study, it was found that the enzymatic activity U of the recombinant protein RpfB was 4.74 nmol/mL, and the lysozyme activity U was 21.52 nmol/mL.

### Effect of temperature on enzyme activity and stability

**Figure 5:**
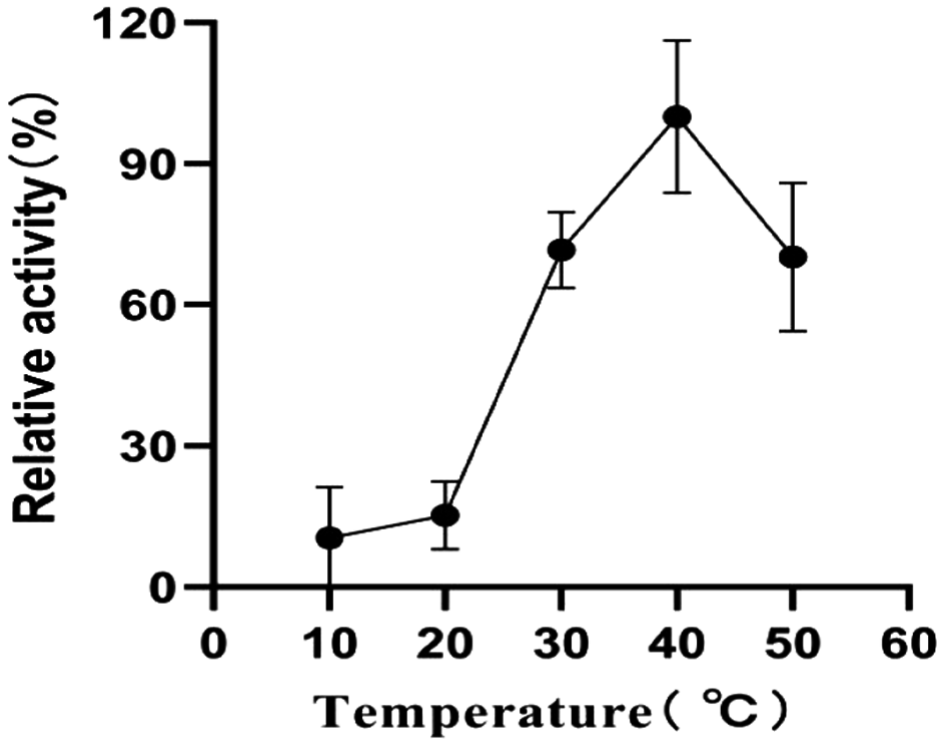
The effect of temperature on the activity of RpfB recombinant protease.

The determined temperature-activity profile of RpfB-GX12401 is shown in Figure 5. RpfB muralytic activity was increased gradually with increasing temperature from 10°C to 50°C; maximum activity was found at 40°C. Relative enzyme activity of >60% was retained at 50°C, but the activity of RpfB decreased markedly above 40°C. Moreover, the lysozyme activity of RpfB protein remained above 50% after 7 h of incubation in lysozyme activity experiment at 10°C ~ 50°C. Therefore, the lysozyme activity of RpfB protein is stable in the range of 10°C–50°C. To sum up, the temperature at which RpfB has the best lysozyme activity is 40°C.

### Effect of chemical reagents, metal ions, and solvents on enzyme activity and stability

**Figure 6:**
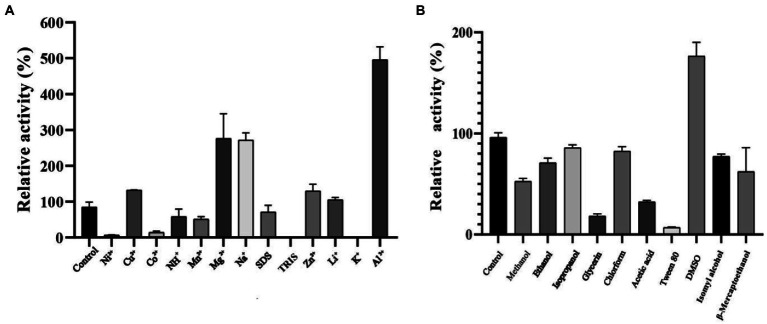
**(A)** Effects of metal ions and chemical reagents on the activity of RpfB recombinant protein. **(B)** Effect of organic solvents on RpfB activity. Untreated enzyme was used as the control and defined as 100% relative activity.

Effect of chemical reagents, metal ions, and solvents on enzyme activity and stability the influence of metal ions and chemical reagents on the activity of purified RpfB is shown in Figure 6A. The presence of Mg^2+^, Na^+^, and Al^3+^ significantly increased the activity of RpfB-GX12401. We found that the activity of RpfB-GX12401 was not significantly affected by Cu^2+^, Zn^2+^, and Li^+^ in the present study. When mixed with 100 mM NH^4+^, Mn^2+^, or SDS, the residual activity of RpfB-GX12401 was high. We found RpfB-GX12401 although the activity dropped sharply when the concentration of Ni^2+^, Co^2+^, and TRIS was 100 mM and K^+^ was 20 mM. We found RpfB activity was strongly inhibited at relative tested concentrations of Ni^2+^, Co^2+^, TRIS, and K^+^ (relative activity of < 60% in each case). The influence of various organic solvents on the stability of purified RpfB is shown in Figure 6B; the activity obviously increased in the presence of DMSO. Most of the organic solvents tested in the present study; including methanol, ethanol, isopropanol, chloroform, isopentyl alcohol, and β-mercaptoethanol; caused RpfB activity to decrease somewhat when used at 1% final concentration. However, glycerin, acetic, and Tween 80 acid strongly inhibited the RpfB activity (by > 50%) at both concentrations.

### Induction and resuscitation of VBNC state of *Rhodococcus* sp. (GX12401)

**Figure 7:**
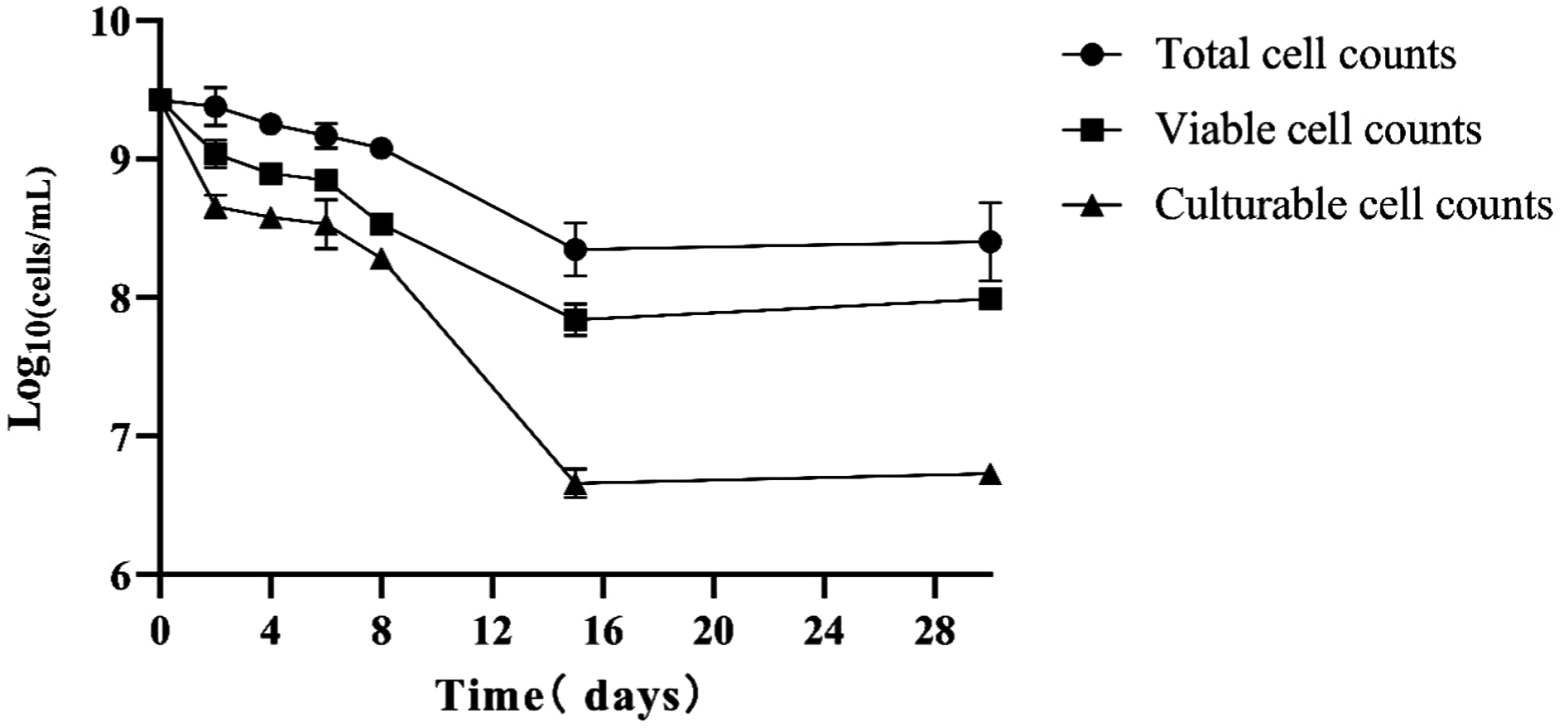
Induction of VBNC state of *Rhodococcus* sp. (GX12401).

**Figure 8:**
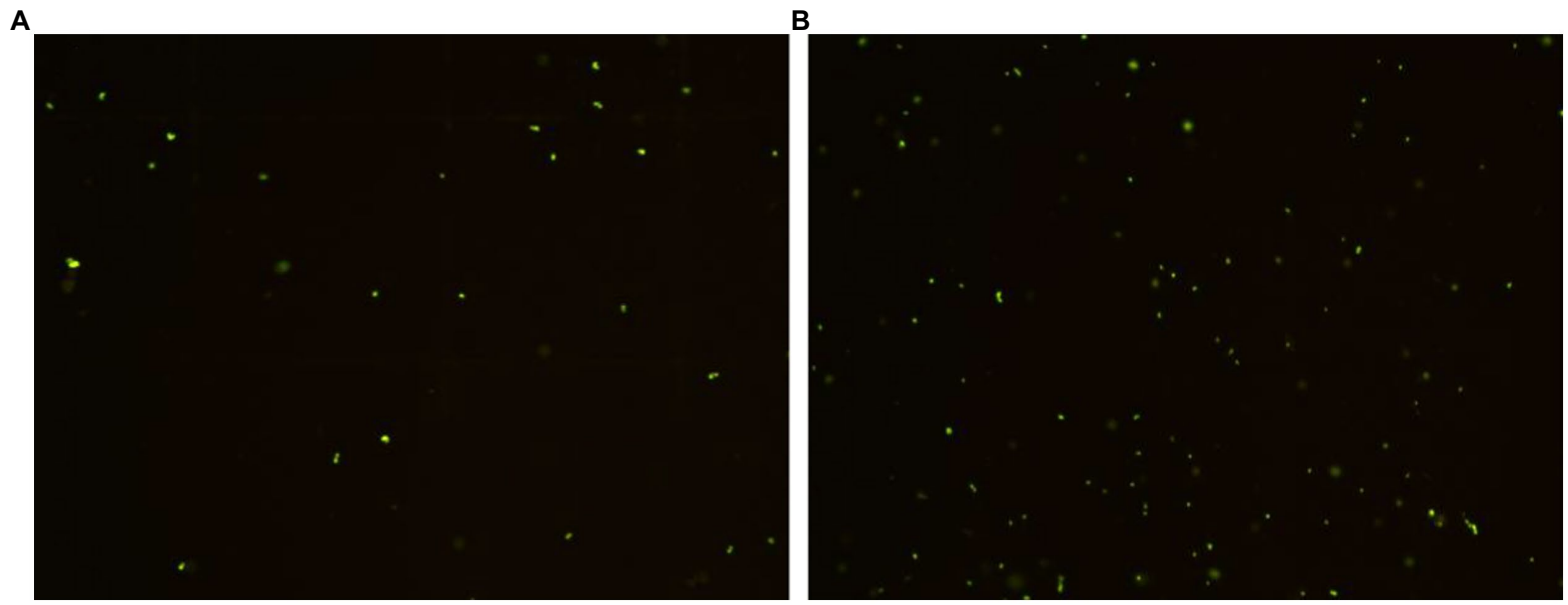
Fluorescence microscopic observation of *Rhodococcus* sp. (GX12401) active phase **(A)** and VBNC phase cells **(B)**. **(A)** Active phase fluorescence field, **(B)** VBNC fluorescence field, 40×.

**Figure 9:**
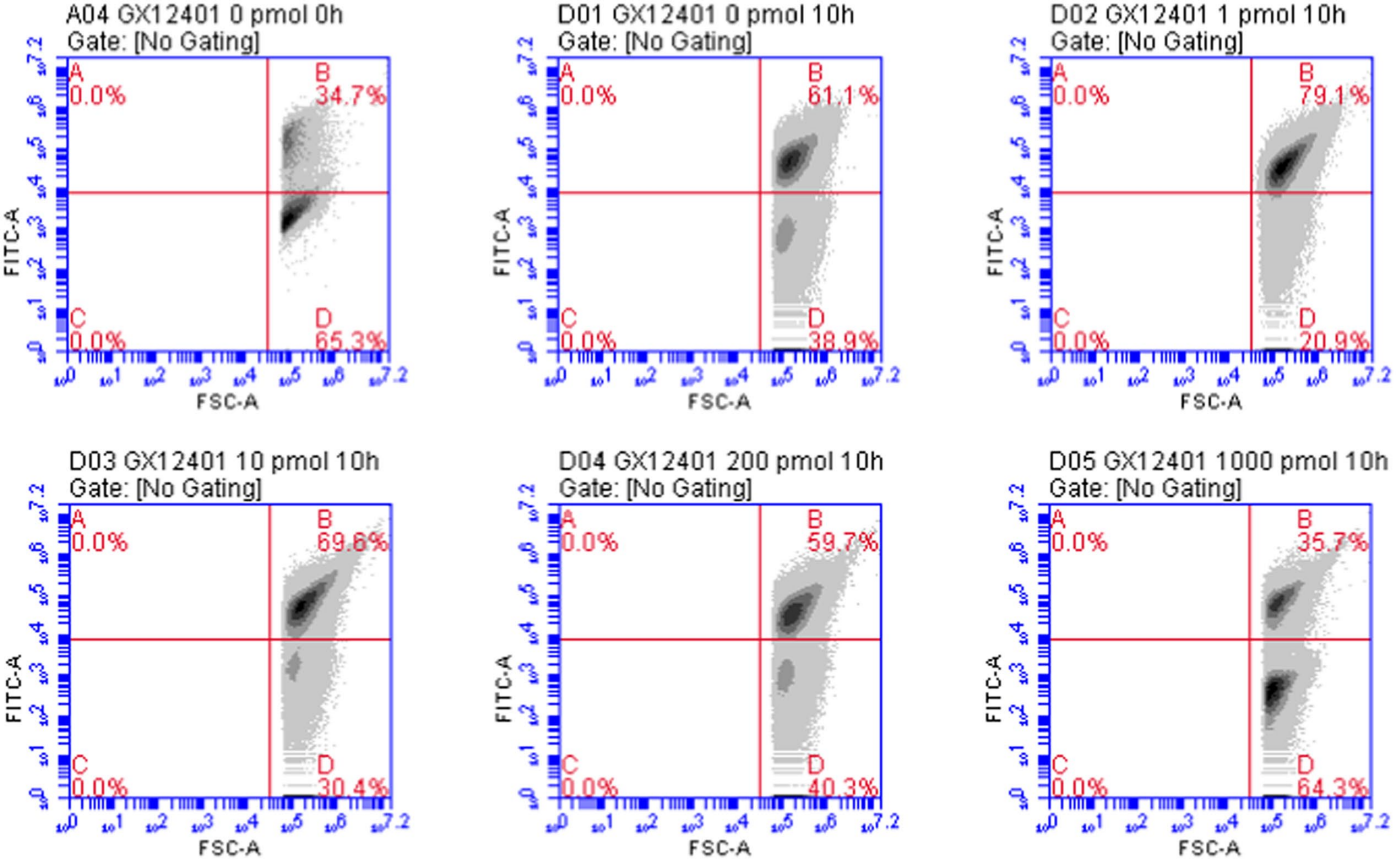
Flow cytometry results map. GX12401: *Rhodococcus* sp. (GX12401) normal cells and VBNC cells, A04: VBNC cells before resuscitation flow analysis map, D01 ~ D05: adding 0 picomolar concentrations, 1 pM concentrations, 10 picomolar concentrations, 200 picomolar concentrations, 1,000 pM concentrations recombinant protein RpfB resuscitation VBNC cell flow analysis map, B region: living cell proportion, D region: dead cell proportion.

*Rhodococcus* sp. (GX12401) VBNC cells were induced by ciprofloxacin, and VBNC cells appeared on the second day, reached the maximum, and stabilized on the 15th day, which provided stable VBNC cells and the time point of RpfB addition for the follow-up experiment, as shown in Figure 7. Tested by API ZYM reagent strip, the results are shown in Table 1. After *Rhodococcus* sp. (GX12401) entered VBNC, the esterase (C4) and esterase lipase (C8) were significantly reduced. Lipase (C14), trypsin, and α-chymotrypsin were slightly elevated. The active phase and VBNC cells of the *Rhodococcus* sp. (GX12401) strain were observed by fluorescent staining (OD600 ≈ 0.2) by CFDA CE reagent, and the fluorescence intensity of the active phase cells was stronger than that of VBNC cells, as shown in Figures 8A,B. Recombinant protein RpfB can promote resuscitation through *Rhodococcus* sp. (GX12401). Different picomolar concentration levels of recombinant protein RpfB have different recovery intensities in *Rhodococcus* sp. (GX12401) VBNC cells. The cells at this stage were stained with CFDA CE dye, and the cells in VBNC state were detected by flow cytometry. The percentage of living cells was 34.7% before induction, as shown in Figure 9 A04. After 10 h of induction, the percentage of living cells without recombinant protein RpfB was 61.1%, while that of living cells with 1,000 picomolar concentrations recombinant protein RpfB was 35.7%, indicating that high concentration of RpfB protein inhibited the growth and recovery of *Rhodococcus* sp. (GX12401) normal cells and VBNC cells, as shown in Figure 9 D01 and D05 below. When the recombinant protein RpfB was added at 1, 10, and 200 pM concentrations, the percentage of living cells was 79.1%, 69.6%, and 59.7%, respectively. It had the best resuscitation activity at 1 pM concentrations, which increased by 18% compared with the control. The growth and recovery of cells was completely inhibited at 1,000 picomolar concentrations, as shown in Figure 9 D02, D03, D04.

**Table 1:** Detection of extracellular enzyme secretion.

Enzyme assayed for	Active state(nM)	VBNC state (nM)	Result
Esterase (C4)	≈ 30	≈ 0	−
Esterase lipase (C8)	≈ 40	≈ 10	−
Lipase (C14)	≈ 5	≈ 10	+
Leucine arylamidase	≈ 40	≈ 40	
Valine arylamidase	≈ 10	≈ 10	
Cystine arylamidase	≈ 10	≈ 10	
Trypsin	≈ 5	≈ 10	+
α-chymotrypsin	≈ 5	≈ 10	+
Acid phosphatase	≈ 30	≈ 30	
Naphthol-AS-BI-phosphohydrolase	≈ 30	≈ 30	
α-glucosidase	≈ 20	≈ 20	
β-glucuronidase	≈ 40	≈ 40	

“+” means that the concentration of extracellular enzymes increases, “–” means that the concentration of extracellular enzymes decreases, and no sign means that the concentration of extracellular enzymes remains unchanged.

## Discussion

RpfB protein is a cell-wall glycosidase, which cleaves cell-wall peptidoglycan (Ruggiero et al., 2011). Among the five known Rpf proteins, RpfB is the most complex protein which consists of three types of domains, including catalytic domain, G5 domain, and three DUF348 domains (Ruggiero et al., 2016). The homologous modeling results of *Rhodococcus* sp. (GX12401) *rpfB* gene showed that the RpfB protein had the highest homology with *M. tuberculosis rpfB*, and the sequence matching degree was 53.66% (<70% was identified as a new protein), which contained a complete domain (Waterhouse et al., 2018). In existing studies, it has been proved that the G5 domain is related to the adsorption function of Rpf protein, and in the crystal structure study of RpfB_3D_, based on its structural similarity with ubiquitin and frequent association with the G5 domain, it was named G5—the linked-ubiquitin-like domain, UBLG5, reveals that the Rpf protein is structurally similar to ubiquitin (Ruggiero et al., 2016). Analysis of the *Rhodococcus* RpfB protein domain showed that the RpfB proteins of different strains of the same genus were different. The *Rhodococcus* sp. (GX12401) protein contained a complete domain, while *R. qingshengii* and *R. hoagii* lacked a DUF348, indicating that the RpfB protein is in the process of species adaptation to the environment. Changes occur, but deletion of the DUF348 structure does not render the RpfB protein inactive, whereas the transglycosylase domain and the G5 domain are possessed by every RpfB, so these two domains are integral to the RpfB protein. In the heterologous expression of genes, the expression of foreign genes will increase the metabolic load of the host bacteria, and the overload will affect the growth of the host bacteria. In the optimization experiment of induced expression of pET-30a(+)-*rpfB-E. coil* BL21 strain, the strain will no longer have the ability of expression when the value of OD_600_ is more than 0.5, but it will have high expression ability when the value of OD_6007_ is between 0.4 and 0.5. This phenomenon may be related to the lysozyme activity of RpfB protein (Mukamolova et al., 2006). After optimizing the conditions, the recombinant protein RpfB was successfully purified by Ni affinity chromatography, indicating that protein was a soluble protein. In the study of the enzymatic properties of the recombinant protein RpfB, Mg^2+^, Na^+^, Al^3+^, Zn^2+^, and DMSO could promote the enzyme, while Ni^2+^, Co^2+^, Tris, K^+^, glycerol, acetate and Tween 80 could inhibit the enzyme. Like the known Rpf protein in *Rhodococcus erythropinus* KB1, Zn^2+^ could promote the activity of both, so adding Zn^2+^ to the resuscitation of *Rhodococcus* could improve the resuscitation activity. The lysozyme activities of egg white lysozyme and RpfB protein were both improved under the catalysis of sodium ions, and Tween 80 inhibited the enzymatic activity of both, so in the follow-up research, it is necessary to avoid the use of Tween 80 (Liu et al., 2008). At the same time, it can be used as an RpfB protein inhibitor to block subsequent enzymatic reactions. In addition, the enzyme activity of recombinant protein RpfB is highly sensitive to chemical reagents, which is easily affected by metal ions and organic compounds in the experimental study of protein function. The cell wall that enters the VBNC state will change (Jia et al., 2020), which not only protects itself but hinders its own recovery, and the Rpf protein with low lysozyme activity ensures the function of cutting the cell wall without complete destroying the cell wall, thus providing suitable conditions environment for the recovery of VBNC cells (Mukamolova et al., 2006). The lysozyme activity of the RpfB protein of *Rhodococcus erythropolis* at 2.07 U (Yue, 2014), while the lysozyme activity of the recombinant protein RpfB purified in this study was 4.74 U, and the lysozyme activity of the reference protein was 21.52 U, which improved the lysozyme activity of the lysozyme, enzyme activity while maintaining low lysozyme activity.

Oligotrophic medium, low temperature, and antibiotics are commonly used ways to induce bacterial entry into VBNC (Su et al., 2015a, 2016). Ciprofloxacin is a third-generation quinolone antibacterial drug with strong antibacterial activity and broad-spectrum (Mishra et al., 2011). Existing studies have shown that it takes at least four-month-old to induce *R. biphenylivorans* TG9^T^ and *M. tuberculosis* H37\Ra to enter the VBNC state using oligotrophic, low temperature (Gao et al., 2007; Su et al., 2015a), using the antibacterial activity of ciprofloxacin to induce *Rhodococcus* sp. (GX12401) VBNC cells, VBNC cells began to appear on the second day, reached a maximum value on the 15th day and reached a stable period, which can significantly shorten the period of entering the VBNC, which is crucial for the subsequent study of resuscitation conditions. After bacteria enter VBNC, the body will reduce metabolism and stop cell division (Xu et al., 2018). The cellular enzyme activities were assessed by using API ZYM kit according to the color intensity of each enzyme. Compared with the exponential phase cells, the esterase (C4) and esterase lipase (C8) activities in VBNC cells of *Rhodococcus* sp. (GX12401) were lower, and the esterase (C4) was mainly involved in lipid hydrolysis and intracellular energy metabolism. Under the continuous inhibition of antibiotics, the activities of trypsin and α-chymotrypsin increased in *Rhodococcus* sp. (GX12401) during VBNC, which was different from that of TG9^T^ VBNC cells only after resuscitation (Su et al., 2015a; Ye et al., 2020). We speculate that the increase in the activity of these two enzymes in VBNC cells is related to the increase in the probability of denatured proteins, and the increase of trypsin and chymotrypsin can help cells to remove denatured proteins and thus help cells maintain normal function. In the fluorescence microscope observation of VBNC cells, the CFDA CE substance is based on the integrity of the cell membrane, so dead cells are not stained (Zhao et al., 2019). *Rhodococcus* sp. (GX12401) cells in the active phase have strong fluorescence intensity because they have a complete cell membrane and synthesize more abundant non-specific esterases, while the fluorescence intensity of VBNC cells is significantly weakened and still shows green fluorescence, indicating that their cell membrane integrity is reduced and non-specific esterase synthesis decreased significantly, which was related to the significant decrease in the expression of esterase (C4) and lipid esterase (C8). Recombinant proteins RpfB are functionally similar to known Rpf proteins, they are biologically active at optimal concentrations, above which they are either inactive or inhibited (Mukamolova et al., 2002). In this study, the most suitable amount of recombinant protein was 1 picomolar concentrations, and when it was higher than 1,000 picomolar concentrations, the growth of the strain was inhibited.

At present, the *rpfs* gene of *Rhodococcus* is the most in-depth research in *Rhodococcus erythropolis*, mainly focusing on the heterologous expression of *rpfs* gene, gene mutation, biodegradation, soil remediation, recovery of VBNC strains, and so on. In addition, by treating the isolated samples with Rpf protein, the isolated strains had efficient degradation performance on reactive blue 19, indicating that *Rhodococcus* played an important role in ecological restoration (Luo et al., 2019; Cai et al., 2021). The microorganisms in the natural environment are characterized by a high degree of diversity, but the limitations of using traditional methods to isolate microorganisms in the natural environment are increasing. The application and research of *Rhodococcus* in the screening of new microorganisms is relatively rare. The existing research shows that the Rpf protein of *Rhodococcus erythropolis* can increase the diversity of culturable oil-degrading bacteria, and the culturable bacterial phyla increased from 9 to 13 and 16, respectively (Fu et al., 2021); in the exploration of VBNC flora with denitrification ability, a total of 13 strains with heterotrophic nitrification ability were obtained after adding Rpf protein (Su et al., 2019). The heterologously expressed RpfB protein in our laboratory has good enzymatic activity and can revive its own VBNC cells, so the application of RpfB protein will provide more possibilities for subsequent screening of new microbial species and functions.

## Conclusion

In this study, a stable potential *Rhodococcus* strain was screened from mangrove sediments, and its resuscitation-promoting factor (RPF) was heterogeneously expressed. The experiment of enzymatic properties showed that the recombinant protein RpfB had good stability, enzyme activity, and low lysozyme activity (4.74 U), in which Mg^2+^, Na^+^, Al^3+^, Zn^2+^, and DMSO could significantly increase the activity of RpfB, and the VBNC state of resuscitation *Rhodococcus* sp. (GX12401) at 1 picomolar concentration concentrations was 18% higher than that of the control group. The above analysis results laid a foundation for the practical application of *Rhodococcus* and its source resuscitation-promoting factors.

## Data availability statement

The data presented in the study are deposited in the NCBI repository, accession number ON357679.

## Author contributions

XG: Conceive and design experiments, conduct experiments, analyze data, prepare graphs and tables, write or review draft papers, and approve final drafts. HL: Conceive and design experiments, conduct experiments, approve final draft, revise article. JW: Conceived and designed experiments, provided experimental instruments, and analyzed data. YZ: Analyze data, review manuscripts, and revise articles. LY: Provide experimental equipment, revise the article and approve the final draft. YW: Analyze proteins and participate in flow cytometry data analysis. NS: Collect experimental samples, analyze and process experimental samples. MJ: Provides laboratory site reagent material analysis tools, methods and formal analysis, resource and funding access. All authors contributed to this article and approved the submitted version.

## Funding

This research was funded by the Major Research Project of Guangxi for Science and Technology (AA18242026) and the Guangxi Natural Science Foundation Project (Gui Ke AB21196020). The National Natural Science Foundation of China (no 81960614) and Innovation Project of GuangXi Minzu University Graduate Educat (gxun-chxs 2021070).

## Conflict of interest

The authors declare that the research was conducted in the absence of any commercial or financial relationships that could be construed as a potential conflict of interest.

## Publisher’s note

All claims expressed in this article are solely those of the authors and do not necessarily represent those of their affiliated organizations, or those of the publisher, the editors and the reviewers. Any product that may be evaluated in this article, or claim that may be made by its manufacturer, is not guaranteed or endorsed by the publisher.

## Footnotes

^1^ https://www.ncbi.nlm.nih.gov/orffifinder/

^2^ https://blast.ncbi.nlm.nih.gov/Blast.cgi

^3^ https://swissmodel.expasy.org/

^4^ https://espript.ibcp.fr/ESPript/cgi-bin/ESPript.cgi

## References

[ref1] Cai J. Pan A. Li Y. Xiao Y. Zhou Y. Chen C.. (2021). A novel strategy for enhancing anaerobic biodegradation of an anthraquinone dye reactive blue 19 with resuscitation-promoting factors. Chemosphere 263:127922. doi: 10.1016/j.chemosphere.2020.127922, PMID: 32841875

[ref2] Dong K. Pan H. Yang D. Rao L. Zhao L. Wang Y.. (2020). Induction, detection, formation, and resuscitation of viable but non-culturable state microorganisms. Compr. Rev. Food Sci. Food Saf. 19, 149–183. doi: 10.1111/1541-4337.12513, PMID: 33319518

[ref3] Fu J. Chen J. Wang Y. Meng T. Yue L. Luo D.. (2021). Promoting effect of the recombinant resuscitation promoting factors-2 of Rhodococcus erythropolis on petroleum degradation and cultivable bacterial diversities of the oil contaminated soils. Lett. Appl. Microbiol. 74, 462–469. doi: 10.1111/lam.13626 34878651

[ref4] Gao H. Bai Y. Xue Y. Wang L. Fan A. Ding X.. (2007). Expression, purification, and characterization of soluble RpfD with high bioactivity as a recombinant protein in mycobacterium vaccae. Protein Expr. Purif. 55, 112–118. doi: 10.1016/j.pep.2007.05.002, PMID: 17576074

[ref5] Hartmann M. Barsch A. Niehaus K. Pühler A. Tauch A. Kalinowski J. (2004). The glycosylated cell surface protein Rpf2, containing a resuscitation-promoting factor motif, is involved in intercellular communication of *Corynebacterium glutamicum*. Arch. Microbiol. 182, 299–312. doi: 10.1007/s00203-004-0713-1, PMID: 15480574

[ref6] Jia Y. Yu C. Fan J. Fu Y. Ye Z. Guo X.. (2020). Alterations in the cell wall of Rhodococcus biphenylivorans under norfloxacin stress. Front. Microbiol. 11:554957. doi: 10.3389/fmicb.2020.554957, PMID: 33123102 PMC7573542

[ref7] Jungwirth B. Emer D. Brune I. Hansmeier N. PÃ¼hler A. Eikmanns B. J.. (2008). Triple transcriptional control of the resuscitation promoting factor 2 (rpf2) gene of Corynebacterium glutamicum by the regulators of acetate metabolism RamA and RamB and the cAMP-dependent regulator GlxR. FEMS Microbiol. Lett. 281, 190–197. doi: 10.1111/j.1574-6968.2008.01098.x, PMID: 18355281

[ref8] Liu H. Wang F. Chu J. (2008). Study on some enzymological properties and activity influencing factors of egg white lysozyme. Chin. J. Biochem. Pharm. 29, 385–387.

[ref9] Luo D. Chen J. Xie G. Yue L. Wang Y. (2019). enzyme characterization and biological activities of a resuscitation promoting factor from an oil degrading bacterium Rhodococcus erythropolis KB1. PeerJ 2019, 1–18. doi: 10.7717/peerj.6951, PMID: 31149404 PMC6534110

[ref10] Mishra A. Taneja N. Sharma M. (2011). Demonstration of viable but nonculturable vibrio cholerae O1 in fresh water environment of India using ciprofloxacin DFA-DVC method. Lett. Appl. Microbiol. 53, 124–126. doi: 10.1111/j.1472-765X.2011.03077.x, PMID: 21554341

[ref11] Mukamolova G. V. Kaprelyants A. S. Young D. I. Young M. Kell D. B. (1998). A bacterial cytokine. Proc. Natl. Acad. Sci. U. S. A. 95, 8916–8921. doi: 10.1073/pnas.95.15.8916, PMID: 9671779 PMC21177

[ref12] Mukamolova G. V. Murzin A. G. Salina E. G. Demina G. R. Kell D. B. Kaprelyants A. S.. (2006). Muralytic activity of Micrococcus luteus Rpf and its relationship to physiological activity in promoting bacterial growth and resuscitation. Mol. Microbiol. 59, 84–98. doi: 10.1111/j.1365-2958.2005.04930.x, PMID: 16359320

[ref13] Mukamolova G. V. Turapov O. A. Young D. I. Kaprelyants A. S. Kell D. B. Young M. (2002). A family of autocrine growth factors in mycobacterium tuberculosis. Mol. Microbiol. 46, 623–635. doi: 10.1046/j.1365-2958.2002.03184.x, PMID: 12410821

[ref14] Punta M. Coggill P. C. Eberhardt R. Y. Mistry J. Tate J. Boursnell C.. (2012). The Pfam protein families database. Nucleic Acids Res. 40, D290–D301. doi: 10.1093/nar/gkr1065, PMID: 22127870 PMC3245129

[ref15] Ravel J. Hill R. T. Colwell R. R. (1994). Isolation of a vibrio cholerae transposon-mutant with an altered viable but nonculturable response. FEMS Microbiol. Lett. 120, 57–61. doi: 10.1111/j.1574-6968.1994.tb07007.x, PMID: 8056295

[ref16] Ruggiero A. Squeglia F. Pirone L. Correale S. Berisio R. (2011). Expression, purification, crystallization and preliminary X-ray crystallographic analysis of a major fragment of the resuscitation-promoting factor Rpf B from *Mycobacterium tuberculosis*. Acta Crystallogr. Sect. F: Struct. Biol. Cryst. Commun. 67, 164–168. doi: 10.1107/S1744309110049845, PMID: 21206053 PMC3080001

[ref17] Ruggiero A. Squeglia F. Romano M. Vitagliano L. de Simone A. Berisio R. (2016). The structure of resuscitation promoting factor B from *M. tuberculosis* reveals unexpected ubiquitin-like domains. BBA-Gen. Subjects 1860, 445–451. doi: 10.1016/j.bbagen.2015.11.001, PMID: 26549874

[ref18] Sexton D. L. St-Onge R. J. Haiser H. J. Yousef M. R. Brady L. Gao C.. (2015). Resuscitation-promoting factors are cell wall-lytic enzymes with important roles in the germination and growth of streptomyces coelicolor. J. Bacteriol. 197, 848–860. doi: 10.1128/JB.02464-14, PMID: 25512314 PMC4325095

[ref19] Shi S. Yang L. Jiang M. (2018). *A Comparison of Actinomycetes Isolation medium with Samples from Mangrove Habitats in Maowei Sea, Guangxi Beibu Gulf, Microbiology*. China: Microbiology, 2331–2340.

[ref20] Su X. Chen X. Hu J. Shen C. Ding L. (2013). Exploring the potential environmental functions of viable but non-culturable bacteria. World J. Microbiol. Biotechnol. 29, 2213–2218. doi: 10.1007/s11274-013-1390-5, PMID: 23733177

[ref21] Su X. Guo L. Ding L. Qu K. Shen C. (2016). Induction of viable but nonculturable state in rhodococcus and transcriptome analysis using RNA-seq. PLoS One 11, 1–19. doi: 10.1371/journal.pone.0147593, PMID: 26808070 PMC4725852

[ref22] Su X. Sun F. Wang Y. Hashmi M. Z. Guo L. Ding L.. (2015a). Identification, characterization and molecular analysis of the viable but nonculturable Rhodococcus biphenylivorans. Sci. Rep. 5, 1–12. doi: 10.1038/srep18590, PMID: 26687808 PMC4685647

[ref23] Su X. Xue B. Wang Y. Hashmi M. Z. Lin H. Chen J.. (2019). Bacterial community shifts evaluation in the sediments of Puyang River and its nitrogen removal capabilities exploration by resuscitation promoting factor. Ecotoxicol. Environ. Saf. 179, 188–197. doi: 10.1016/j.ecoenv.2019.04.067, PMID: 31048215

[ref24] Su X. Zhang Q. Hu J. Hashmi M. Z. Ding L. Shen C. (2015b). Enhanced degradation of biphenyl from PCB-contaminated sediments: the impact of extracellular organic matter from *Micrococcus luteus*. Appl. Microbiol. Biotechnol. 99, 1989–2000. doi: 10.1007/s00253-014-6108-6, PMID: 25301582

[ref25] Su X. Zhang S. Mei R. Zhang Y. Hashmi M. Z. Liu J.. (2018). Resuscitation of viable but non-culturable bacteria to enhance the cellulose-degrading capability of bacterial community in composting. Microb. Biotechnol. 11, 527–536. doi: 10.1111/1751-7915.13256, PMID: 29536669 PMC5902322

[ref500] Tang W. Zhang J. Wang Z. Hong M. (2000). Reasons for deviation in molecular weight of His-tag fusion protein determined by SDS-PAGE method. J. Plant Physiol. 0257-4829, 64–68.

[ref26] Telkov M. V. Demina G. R. Voloshin S. A. Salina E. G. Dudik T. V. Stekhanova T. N.. (2006). Proteins of the Rpf (resuscitation promoting factor) family are peptidoglycan hydrolases. Biochem. Mosc. 71, 414–422. doi: 10.1134/S0006297906040092, PMID: 16615861

[ref27] Tufariello J. A. M. Mi K. Xu J. Manabe Y. C. Kesavan A. K. Drumm J.. (2006). Deletion of the mycobacterium tuberculosis resuscitation-promoting factor Rv1009 gene results in delayed reactivation from chronic tuberculosis. Infect. Immun. 74, 2985–2995. doi: 10.1128/IAI.74.5.2985-2995.2006, PMID: 16622237 PMC1459759

[ref28] Waterhouse A. Bertoni M. Bienert S. Studer G. Tauriello G. Gumienny R.. (2018). SWISS-MODEL: homology modelling of protein structures and complexes. Nucleic Acids Res. 46, W296–W303. doi: 10.1093/nar/gky427, PMID: 29788355 PMC6030848

[ref29] Xu T. Cao H. Zhu W. Wang M. du Y. Yin Z.. (2018). RNA-seq-based monitoring of gene expression changes of viable but non-culturable state of vibrio cholerae induced by cold seawater. Environ. Microbiol. Rep. 10, 594–604. doi: 10.1111/1758-2229.12685, PMID: 30058121

[ref30] Xu H. Roberts N. Singleton F. Attwell R. Grimes J. Colwell R. (1982). Survival and viability of nonculturable *Escherichia coli* and vibrio cholerae in the estuarine and marine environment. Microb. Ecol. 8, 313–323. doi: 10.1007/BF02010671, PMID: 24226049

[ref31] Ye Z. Li H. Jia Y. Fan J. Wan J. Guo L.. (2020). Supplementing resuscitation-promoting factor (Rpf) enhanced biodegradation of polychlorinated biphenyls (PCBs) by Rhodococcus biphenylivorans strain TG9T. Environ. Pollut. 263:114488. doi: 10.1016/j.envpol.2020.114488, PMID: 32244156

[ref32] Yue L. (2014). Studies on gene cloning, expression and biological characterization of the resuscitation-promoting factor of *Rhodococcus erythropolis*. master's thesis [China (GS)]: Lanzhou University of Technology.

[ref33] Zhao X. Li M. Tang J. Zou H. Chen G. Zhang J.. (2019). “Quantitative analysis of colonization of *Lactobacillus plantarum* RS-09 in gastrointestinal tract by fluorescence labeling.” in *World Conference on Animal Welfare Science*; May 28, 2019; 258–268.

[ref34] Zhu W. Plikaytis B. B. Shinnick T. M. (2003). Resuscitation factors from mycobacteria: homologs of Micrococcus luteus proteins. Tuberculosis 83, 261–269. doi: 10.1016/S1472-9792(03)00052-0, PMID: 12906837

