# Supplementary figures and images for: Enzymatic properties and biological activity of resuscitation-promoting factor B of Rhodococcus sp. (GX12401)
Source: Front Microbiol. 2022 Oct 5;13:965843. doi: 10.3389/fmicb.2022.965843 (PMC9580463; doi:10.3389/fmicb.2022.965843)

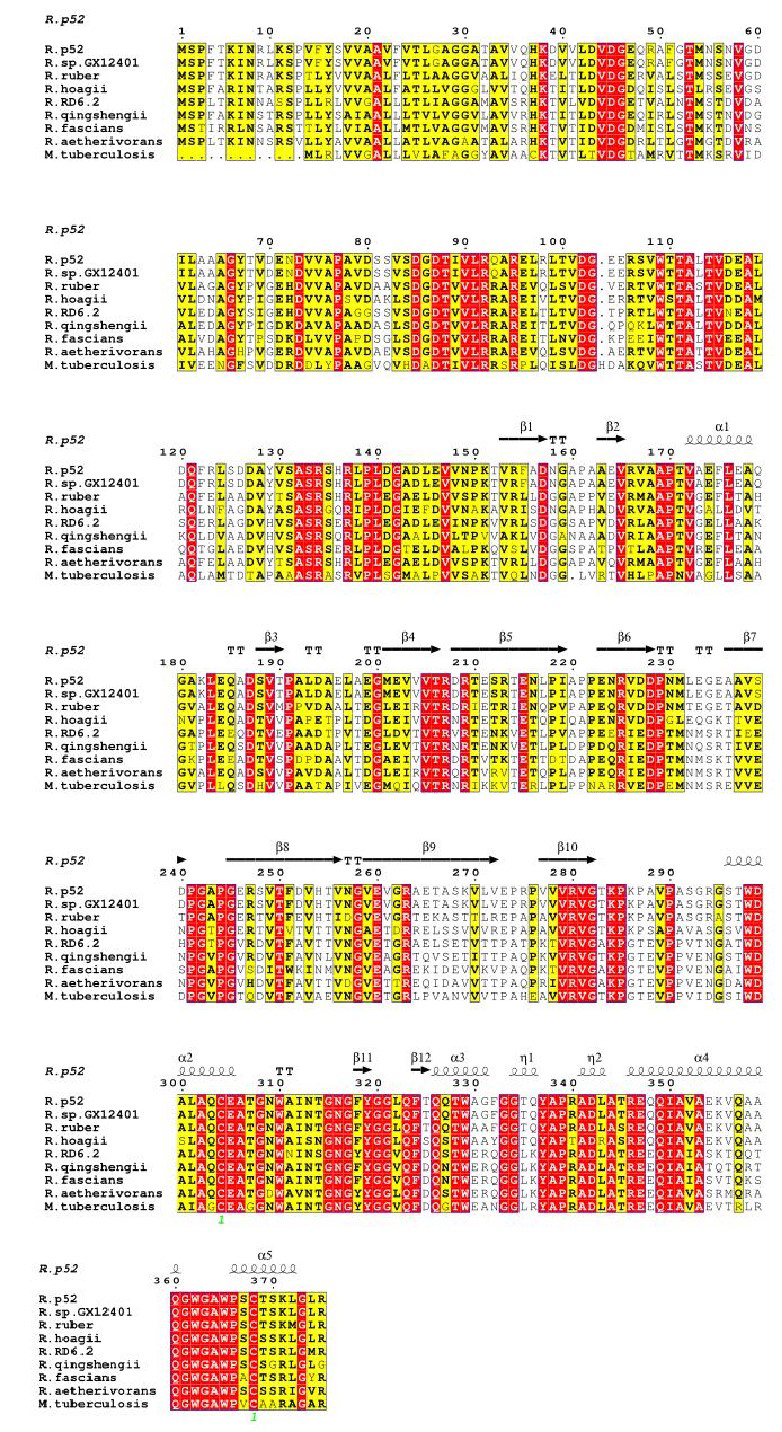

Supplement: Supplementary file 1 [file Image_1.JPEG]

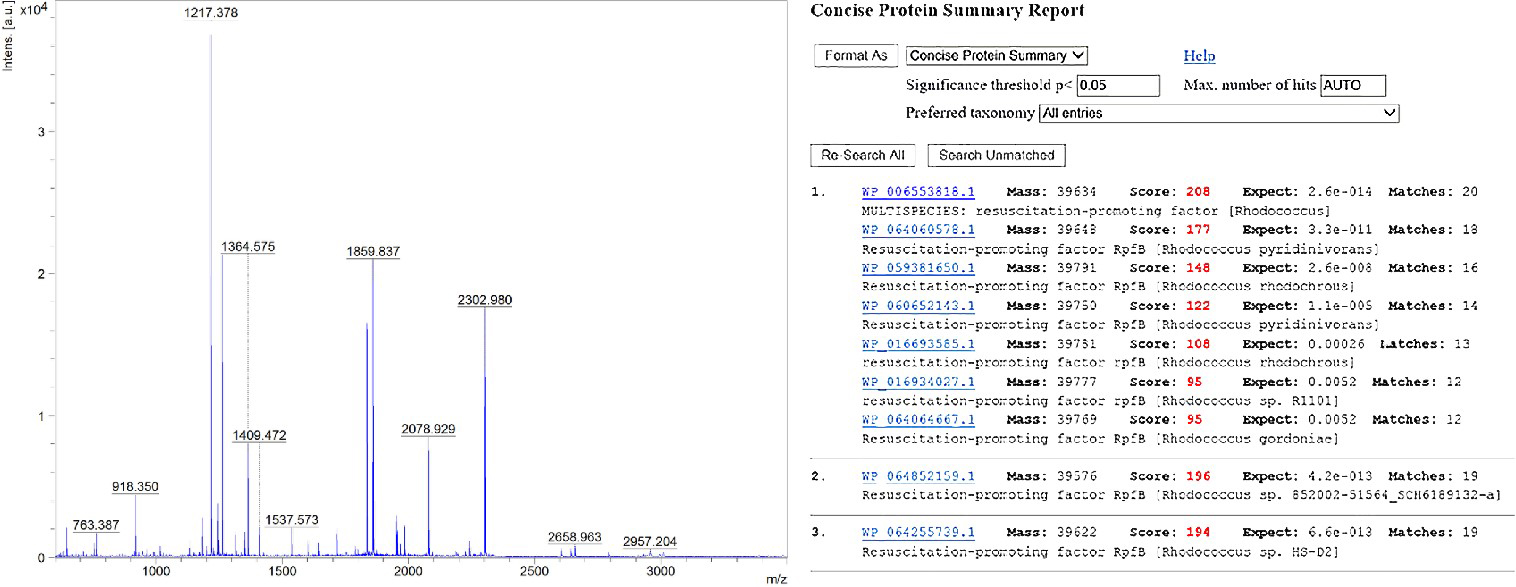

Supplement: Supplementary file 2 [file Image_2.JPEG]
